# Commentary: Non-invasive Brain Stimulation, a Tool to Revert Maladaptive Plasticity in Neuropathic Pain

**DOI:** 10.3389/fnhum.2017.00172

**Published:** 2017-04-07

**Authors:** Arman Taheri, Mahbod Lajevardi, Sara Emami, Sanaz Shabani, Hassan Sharifi

**Affiliations:** ^1^Department of Anesthesiology and Pain Medicine, Imam Khomeini Hospital, Tehran University of Medical SciencesTehran, Iran; ^2^Department of Medical Surgical Nursing, School of Nursing and Midwifery, Iranshahr University of Medical SciencesIranshahr, Iran; ^3^School of Nursing and Midwifery, Mashhad University of Medical SciencesMashhad, Iran

**Keywords:** neuroplasticity, chronic pain, intermittent fasting, brain stimulation, maladaptive plasticity

Pain as a plastic neurophysiological process is a multifaceted sensory and emotional experience. Recent investigations have demonstrated that the neuroplastic changes occur in the structure and function of the brain, particularly in affective and somatosensory regions, in response to chronic pain. These neuroplastic changes could contribute to the maladaptive plasticity (Apkarian et al., 2011); however, recent evidence suggests that these changes may be modifiable and in some cases reversible with specific targeted interventions (Bushnell et al., 2013). In this regard, several promising interventions such as behavioral interventions, human brain stimulation (HBS), feedback and pharmacological interventions were identified for promoting adaptive neuroplastic changes (Cramer et al., 2011).

HBS has been proven effective for treatment of several chronic painful conditions by improving brain function and/or disrupting its activity. Depolarizing neurons, triggering action potentials and changing the brain cortex excitability are among the most important underlying mechanisms of HBS (Davis and van Koningsbruggen, 2013). In their comprehensive and detailed paper regarding non-invasive brain stimulation (NIBS), Naro et al. (2016) argued that NIBS is an effective treatment for preventing and possibly reverting maladaptive plasticity. They suggested that NIBS could stimulate the several cortical areas of the brain and disrupt the ongoing of plastic changes in cortical and sub-cortical structures of the pain matrix.

We would like to congratulate the authors for their exhaustive review. We want to highlight the promising effects of intermittent fasting for improving adaptive neuroplastic changes. In addition, we want to propound the idea of combining NIBS and intermittent fasting as a promising multifaceted therapeutic approach for preventing or possibly reverting the maladaptive plasticity induced by chronic pain.

The efficacy of fasting in reducing weight, delaying aging, and enhancing health status as well as diminishing pain in rheumatoid arthritis is well-documented (Longo and Mattson, 2014). From the perspective of molecular mechanisms, fasting or food deprivation challenges brain function to manage energy efficiently. It has been argued that intermittent fasting may enhance brain function by improving synaptic plasticity, promoting neurogenesis (Longo and Mattson, 2014), enhancing neuronal stress resistance, increasing synaptogenesis, reducing inflammation, promoting motor and cognitive function, as well as exerting complex integrated adaptive responses in the brain and enhancing resistance of the brain to injury (Van Praag et al., 2014). According to Van Praag et al. (2014), fasting affects brain areas by changing the expression of genes that encode proteins involved in “*synaptic plasticity, neurotrophic factor signaling, and cellular bioenergetics, disposal of damaged proteins and organelles, and cellular stress resistance.”*

From the perspective of pathophysiological mechanisms, the most recent evidence suggests that intermittent fasting could improve chronic pain induced neuroplastic changes (Sibille et al., 2016). According to Sibille et al. (2016), the intermittent fasting and administration of glucose could improve cognitive function, neuroplasticity and activate pain-associated paths in brain and ultimately could increase pain treatment effectiveness. In addition, some animals and humans studies have acknowledged that intermittent fasting could prevent brain dysfunction, improve cognitive function and enhance neuroplasticity (Messier, 2004; Fusco and Pani, 2013).

In summary, available evidence supports the benefits of both NIBS and intermittent fasting for preventing and possibly reverting the maladaptive plasticity in chronic neuropathic painful conditions. Therefore, one question has been raised that merits further attention: Could the addition of intermittent fasting improve the effectiveness of NIBS in preventing, reducing and/or reverting the maladaptive plasticity in patients with chronic pain in comparison to each approach separately?

Considering all of aforementioned evidence, it seems that combining brain stimulation and intermittent fasting appear to be promising strategy for improving adaptive neuroplastic changes. This combination has potential to open new windows for better treatment of chronic pain. Therefore, future well-designed studies will be required to confirm the benefit and clinical efficacy of combining brain stimulation and intermittent fasting in preventing or possibly reverting the development of maladaptive brain plasticity in patients with chronic neuropathic pain.

## Author contributions

All of the authors declare that they have all participated in the writing of the paper, and that they have approved the final version.

### Conflict of interest statement

The authors declare that the research was conducted in the absence of any commercial or financial relationships that could be construed as a potential conflict of interest. The reviewer ME and handling Editor declared their shared affiliation, and the handling Editor states that the process nevertheless met the standards of a fair and objective review.
